# Who is the Treatment-Seeking Young Adult with Severe Obesity: A Comprehensive Characterization with Emphasis on Mental Health

**DOI:** 10.1371/journal.pone.0145273

**Published:** 2015-12-22

**Authors:** Helena Dreber, Signy Reynisdottir, Bo Angelin, Erik Hemmingsson

**Affiliations:** 1 Obesity Center, Karolinska University Hospital, Department of Medicine, Karolinska Institutet, Stockholm, Sweden; 2 Department of Medicine, Karolinska Institutet, Stockholm, Sweden; Vanderbilt University, UNITED STATES

## Abstract

**Objective:**

To characterize treatment-seeking young adults (16–25 years) with severe obesity, particularly mental health problems.

**Study Design and Participants:**

Cross-sectional study of 165 participants (132 women, 33 men) with BMI ≥35 kg/m^2^ or ≥30 kg/m^2^ with comorbidities, enrolling in a multidisciplinary obesity treatment program.

**Method:**

Data collection at admission of present and life-time health issues including symptomatology of anxiety, depression (Hospital Anxiety and Depression Scale) and attention-deficit/hyperactivity disorder (Adult ADHD Self-Report scale); self-esteem (Rosenberg Self-Esteem Scale), suicide attempts, health-related quality of life (Short Form-36 Health Survey), psychosocial functioning related to obesity (Obesity-related Problems Scale), cardiorespiratory fitness (Astrand´s bicycle ergometer test), somatic and psychiatric co-morbidities, cardiometabolic risk factors, and micronutritional status. We used multiple regression analysis to identify variables independently associated with present anxiety and depressive symptomatology.

**Results:**

Mean body mass index was 39.2 kg/m^2^ (SD = 5.2). We found evidence of poor mental health, including present psychiatric diagnoses (29%), symptomatology of anxiety (47%), depression (27%) and attention-deficit/hyperactivity disorder (37%); low self-esteem (42%), attempted suicide (12%), and low quality of life (physical component score = 46, SD = 11.2; mental component score = 36, SD = 13.9, P<0.001 for difference). Variables independently associated with present anxiety symptomatology (*R* ^2^ = 0.33, P<0.001) included low self-esteem (P<0.001) and pain (P = 0.003), whereas present depressive symptomatology (*R* ^2^ = 0.38, P<0.001) was independently associated with low self-esteem (P<0.001), low cardiorespiratory fitness (P = 0.009) and obesity-related problems (P = 0.018). The prevalence of type 2 diabetes was 3%, and hypertension 2%. Insulin resistance was present in 82%, lipid abnormality in 62%, and poor cardiorespiratory fitness in 92%. Forty-eight percent had at least one micronutritional deficiency, vitamin D being the most common (35%).

**Conclusion:**

A wide range of health issues, including quite severe mental health problems, was prevalent in treatment-seeking young adults with severe obesity. These are likely to constitute a major treatment challenge, including options relating to bariatric surgery.

## Introduction

Obesity (body mass index ≥30 kg/m^2^) is a global health threat associated with reduced life expectancy and quality of life [1, 2]. In developed countries, the most rapid weight gain occurs in young adulthood with prevalence rates of 8.3% in women and 8.9% in men for 15–19 year-olds, and 13.2% in women and 12.2% in men for 20–24 year-olds (extracted data) [3]. Obesity among young adults is associated with negative metabolic, social and economical consequences in later adulthood [4, 5].

Young adulthood is not defined by chronological age but is characterized sociologically by a transformation to independence, identity building and establishment of behaviors that last into adulthood [6]; and neurologically by a reward-seeking and risk-taking behavior [7].

Evidence suggests that young adulthood is a vulnerable period for weight gain [8, 9], possibly due to young adults´ susceptibility to food marketing as well as to social influences on meal patterns [9, 10]. Lifestyle change programs for young adults have proven challenging [11] while bariatric surgery may result in favorable short-term weight reductions [12]. However, mental health challenges post-bariatric surgery, such as alcoholism and suicide [13–15], cause concerns especially for young adults since the incidence of psychiatric disorders peak at 24–27 years of age [16].

Despite the importance of young adults in the obesity epidemic, this population has been overlooked in clinical obesity research [9], resulting in limited knowledge for clinicians.

Previous clinical characterizations of young adults with obesity have mainly focused on physical health and/or included only pre-bariatric surgery patients or a limited number of participants [17–19], showing higher prevalence rates of cardiometabolic risk factors and psychiatric diagnoses compared to the general population.

Our primary aim was to comprehensively characterize treatment-seeking young adults (16–25 years) with severe obesity, with a specific focus on mental health in order to provide the practitioner with clinical data on an understudied but vulnerable patient category. We also aimed to clarify factors associated with present anxiety and depressive symptomatology in young adults with obesity, independently of body mass index.

## Method

### Subjects and recruitment

This cross-sectional study was conducted at the multidisciplinary young adult section of the Obesity Center at Karolinska University Hospital, serving all municipalities of Stockholm. Patients are referred from outside medical specialists, dietitians or school nurses. Referral requirements to the young adult section of the obesity center are age 16–25 years (definition of young adulthood by Stockholm health care), body mass index (BMI) ≥35 kg/m^2^ or BMI ≥30 kg/m^2^ with obesity-related co-morbidities (somatic or psychiatric). Patients with known eating disorders according to DSM-IV criteria [20], are referred directly to eating disorder specialist clinics for treatment prior to programme initiation at the Obesity Center. Treatment options at the Obesity Center include behavioral management programme or pre-bariatric surgery care.

Patients referred to the young adult section of the Obesity Center, were invited to participate in the study following oral information at their first visit to the clinic. Exclusion criteria: patients in need of a language interpreter or with known diagnosis of an intellectual disability, i.e. patients who were not able to fill in questionnaires independently. Eighty-seven percent (n = 236) out of 270 patients accepted for clinic enrollment met the study inclusion criteria. Seventy percent (n = 165) of eligible patients chose to participate, and provided written and oral consent. For participants <18y, parents’ written consent was obtained.

At the first visit to the Obesity Center, participants were given a self-administered questionnaire to fill in at home, were referred for blood chemistry testing and invited to an enrolment day. On the enrolment day (second visit) the participants were interviewed and examined by a team of obesity professionals. Data from questionnaires, blood tests and clinical interviews/examinations were checked by a physician. In total, the participants visited the Obesity Center twice as part of the study.

The study, including consent providal, was approved by the Stockholm Regional Ethical Review Board (2012/1154-31/4). All data were collected September 2012 –November 2014.

### Clinical data collection

Anthropometry in light clothing and without shoes was assessed by a trained nurse. Body weight was measured to the nearest 0.1 kg using a digital calibrated scale. Height was measured to the nearest 0.5 cm using a wall-mounted stadiometer. BMI was calculated as weight/height^2^ [21]. For participants <18y, class I obesity was calculated according to the definition by Cole et al [22], and class II obesity was calculated according to Kelly et al [23]. Class III obesity was defined as BMI ≥40 kg/m^2^. For participants 18–25 years old, standard World Health Organization BMI categories were used [24].

Waist circumference was measured to the nearest 0.5 cm at the mid-point between the lower margin of the lowest rib and the top of the iliac crest. Blood pressure was assessed once after five minutes rest in sitting position using a manual instrument (Boso manometer, Henry Eriksson AB, Stockholm/Jungingen).

Cardiorespiratory fitness was measured by Astrand´s submaximal bicycle ergometer test for the prediction of VO_2_max [25]. The results (ml VO_2_/kg*min) were classified as either very poor, poor, average or fair; taking gender and age into account in the calculation [25].

A semi-structured interview covering present and life-time incidence of obesity-related comorbidities, medications and treatments; and psychosocial problems was conducted by a physician. Co-morbidities were reported if present in the referral notes or the patient health record (coded according to International Statistical Classification of Diseases and Related Health Problems–Tenth Revision–SE [26]). Medications were classified according to the Anatomic Therapeutic Chemical classification system [27].

Adverse childhood experiences were classified independently by three physicians into no adverse event, absent parent (physical), family history of substance abuse, act of omission (failure to provide for a child´s basic needs) and/or act of commission (words or overt actions that cause harm) [28]. Identical classification by all three doctors was interpreted as a case.

### Questionnaires/self-assessment

The form consisted of widely used questionnaires and single questions. Upon completion, the questionnaire was checked twice together with the participant, firstly by a nurse and secondly by a physician.

The main assessment of mental health problems was symptoms of anxiety and depression, which were examined by using the Hospital Anxiety and Depression Scale (HADS) [29]. HADS is a validated 14-item likert scale used for detection of both anxiety and depression in adults and adolescents [30]. Items are scored 0–3 points and summarized into a continuous variable or categories as follows: no impairment (≤7 points), subclinical (8–10) or clinical impairment (≥11 points) [29].

The Rosenberg Self-Esteem scale [31] was used to detect low self-esteem. The scale includes 10 items, with a four-point likert scale (0–3 points) for scoring, and has been validated in young adults [31, 32]. Low self-esteem was defined as ≤15 points according to the most commonly used cut-off value.

Psychosocial functioning related to obesity was evaluated by using Obesity-related Problems Scale (ver. 1.2), which has been validated in ≥18y and used in ≥13y [33, 34]. The questionnaire comprises eight questions on a four-point likert scale and measures the degree to which participants currently experience obesity-related botherings during daily activities, such as when swimming or socializing. The scores were summarized and transformed into a score of 0–100. A score of <40 points indicates mild, 40–59 points moderate, and ≥60 points severe impairment in obesity-related psychosocial functioning [33].

Health-related quality of life was assessed using the Short Form-36 Health Survey (version 1.0) (SF-36) [35], which has been validated in Swedish 15–93 year-olds [36]. SF-36 is composed of 36 questions about functional health in the last four weeks and the responses are transformed into eight domains and two summary measures (a mental and a physical component score), each ranging from 0 to 100. A score of 100% equals optimal health. The non-norm based scoring system was used in the present study.

Part A of the Adult ADHD Self-Report Scale [37] was used to assess concentration and hyperactivity associated with attention-deficit/hyperactivity disorder (ADHD). The scale was constructed for ≥16y and has been validated in ≥18y [38]. Responses are summarized into a score of 0–6 points. Four points or more is associated with ADHD disease.

Suicide attempt was assessed by a question that had been used and tested in the Swedish National Health Survey [39]: “Have you ever tried to commit suicide”, with the response options of “never”, “yes, more than one year ago”, “yes, past year” or “yes, past week”.

Sleep impairment was assessed by the Karolinska Sleep Questionnaire [40]. The questionnaire is validated in ≥18y and consists of 18 items that are calculated into four subscales of a mean score of 0–5 points: sleep quality, non-restorative sleep, sleep apnea and sleepiness. High values indicate more impairment in the last three months [40]. Prevalence of insomnia was calculated by combining items which correspond to the DSM-IV-criteria [41].

Separate questions were included to assess economic strain, nationality, occupation, pain as measured by EuroQol 5-dimensions (reported as mild or severe) [42], daily tobacco use, alcohol and cannabis use, sexuality, social support, and skin type as measured by Fitzpatrick scale [43]. Apart from the latter, the separate questions followed the same outline as in the Swedish National Health survey [39]. See table in **S1 Table** for further descriptions.

### Metabolic risk factors and nutritional status

Blood samples of metabolic risk factors and micronutritional status were obtained by venous puncture after overnight fasting. See table in **S2 Table** for specification of the included blood samples and threshold values. Insulin resistance was calculated by using the homeostasis model assessment (HOMA-index) [44].

### Statistical analyses

In the classification of glucose, HbA1c and insulin resistance, participants with diabetes mellitus type 1 or 2 were included in the analyses but treated as a separate category.

Participants with prescribed medication for hyperlipidemia or a micronutritional deficiency were classified as a case/deficiency of the corresponding disease/deficiency. Patients with known non-alcoholic fatty liver disease or other known liver diseases were not included in the calculation of frequency of patients with pathological alanine transferase values.

Independent samples t-test was used to compare BMI between genders. Paired samples t-test was used to quantify differences between mental health component scores and physical health component scores. We used multiple regression analysis to identify variables with an independent association to present anxiety and depressive symptomatology, respectively. Known associated variables in the general population were included as covariates in the final models. Non-continuous nominal or ordinal data variables were transformed into binary dummy variables before inclusion in the model.

We included all variables that were significant for either anxiety or depressive symptomatology into the final regression model to be able to compare results across the two main dimensions of mental health. Significant continuous variables from the multivariate analysis were categorized into quartiles, and analyzed in a univariate model to provide estimated marginal means. A p-value <0.05 was considered statistically significant. All analyses were conducted using IBM SPSS statistics version 22.

## Results

Mean BMI was 39.2 kg/m^2^ (SD = 5.2), and 82% (n = 135) were severely obese (≥class II obesity). Twenty-three percent (n = 46) were <18y. Twenty percent were male, and there was no gender difference in BMI (p = 0.25). **Table 1**displays the participants´ anthropometric, sociodemographic and lifestyle characteristics.

**Table 1:** Anthropometric, sociodemographic and lifestyle characteristics in n = 165 treatment-seeking young adults (16–25 y) with obesity.

Characteristic	Women (n = 132)	Men (n = 33)	Total (n = 165)
**Anthropometric measurements**			
Age, y; mean (SD)	19.6 (2.7)	20.2 (2.8)	19.7 (2.7)
Weight, kg; mean (SD)	107.0 (14.3)	133.9 (27.0)	112.4 (20.6)
Body mass index, kg/m^2^; mean (SD)	38.9 (4.9)	40.3 (6.2)	39.2 (5.2)
Body mass index classification; mean (SD)			
*Class I;* n (%)	23 (17)	5 (15)	28 (17)
*Class II;* n (%)	59 (45)	9 (27)	68 (41)
*Class III;* n (%)	49 (37)	18 (55)	67 (41)
Waist circumference, cm; mean (SD)	108.5 (10.3)	126.7 (15.1)	112.1 (13.5)
**Occupation:**			
*Student;* n (%)	83 (63)	23 (70)	106 (64)
*Employed;* n (%)	30 (23)	3 (9)	33 (20)
*Unemployed;* n (%)	11 (8)	6 (18)	17 (10)
*On sickness benefit (>30 consecutive days);* n (%)	5 (4)	0 (0)	5 (3)
**Sickness benefit >30 days last year** *;* n (%)	14 (11)	6 (19)	20 (12)
**Economic strain last year** *;* n (%)	24 (18)	7 (21)	31 (19)
**Place of birth:**			
*Sweden;* n (%)	113 (86)	30 (91)	143 (87)
*Europe outside Sweden;* n (%)	3 (2)	1 (3)	4 (2)
*Africa;* n (%)	3 (2)	0 (0)	3 (2)
*Middle East;* n (%)	7 (5)	2 (6)	9 (6)
*Asia (apart from Middle East);* n (%)	3 (2)	0 (0)	3 (2)
*South America;* n (%)	3 (2)	0 (0)	3 (2)
**Second generation immigrant to Sweden** *;* n (%) ^**a**^	24 (18)	7 (21)	31 (19)
**Lifestyle factors**			
Cardiorespiratory fitness, ml/kg/min; mean (SD) ^b^	24.9 (5.2)	23.0 (5.4)	24.5 (5.3)
*Very poor;* n (%)	57 (72)	19 (100)	76 (78)
*Poor;* n (%)	14 (18)	0 (0)	14 (14)
*Average;* n (%)	5 (6)	0 (0)	5 (5)
*Fair;* n (%)	3 (4)	0 (0)	3 (3)
Daily tobacco smoker*;* n (%)	28 (21)	8 (24)	36 (22)
Cannabis, ever used*;* n (%)	20 (15)	6 (18)	26 (16)
Alcohol, units/week; median (IQR) ^c^	1.0 (0.0–4.0)	0.5 (0.0–3.5)	1.0 (0.0–3.8)
Alcohol, hazardous drinking*;* n (%) ^d^	25 (19)	2 (6)	27 (17)
Karolinska Sleep Questionnaire [40]			
*Sleep quality*; mean (SD)	1.7 (1.2)	1.7 (1.2)	1.7 (1.2)
*Non-restorative sleep*; mean (SD)	2.3 (1.4)	2.4 (1.4)	2.3 (1.4)
*Sleep apnea*; mean (SD)	0.6 (0.9)	0.5 (0.7)	0.6 (0.9)
*Sleepiness*; mean (SD)	1.6 (1.2)	1.5 (1.0)	1.6 (1.2)
*Insomnia;* n (%)	69 (52)	19 (58)	88 (54)
**Sexuality, heterosexual** *;* n (%)	108 (83)	27 (82)	135 (83)

^a^ Both parents born outside Sweden and young adult born within Sweden.

^b^ Astrand’s test [25]. n_women_ = 79–88, n_men_ = 19–22.

^c^ One unit = 12 g of 100% alcohol [45].

^d^ Weekly consumption of 14 units for men and 9 units for women, or consumption of 5 units for men and 4 units for women at the same occasion, according to Swedish criteria [45].

### Cardiometabolic risk factors, somatic disorders and micronutritional deficiencies

**Table 2**displays frequencies of cardiometabolic risk factors, somatic disorders and micronutritional deficiencies. Three percent (n = 4) had HbA1c-values indicative of increased risk of diabetes mellitus and 82% (n = 129) were insulin resistant as defined by elevated HOMA-index for age. Sixty-two percent (n = 98) had at least one plasma lipid abnormality, indicating increased risk for cardiovascular disease, whereof high density lipoprotein-cholesterol below cut-off was the most common (46%, n = 75). Twenty-two percent (n = 35) had elevated alanine aminotransferase values.

**Table 2:** Cardiometabolic risk factors, somatic disorders and micronutritional deficiencies in n = 165 treatment-seeking young adults (16–25 y) with obesity.

Variable	Women (n = 132)	Men (n = 33)	Total (n = 165)
**Cardiometabolic risk factors**			
Fasting plasma-glucose, mmol/l; mean (SD)	5.4 (1.6)	5.3 (0.8)	5.4 (1.5)
HbA1c, mmol/mol; mean (SD)	35.4 (7.4)	33.9 (5.7)	35.1 (7.1)
Insulin, mlU/l; mean (SD)	26.1 (22.4)	28.7 (13.3)	26.6 (20.9)
Total cholesterol, mmol/l; mean (SD)	4.5 (0.9)	4.6 (0.7)	4.5 (0.9)
LDL cholesterol, mmol/l; mean (SD)	2.8 (0.7)	2.9 (0.7)	2.8 (0.7)
HDL cholesterol, mmol/l; mean (SD)	1.1 (0.2)	1.0 (0.2)	1.1 (0.2)
Fasting triglycerides, mmol/l; mean (SD)	1.2 (0.6)	1.6 (0.8)	1.3 (0.6)
ALT, μkat/L; mean (SD)	0.4 (0.3)	1.0 (0.6)	0.5 (0.5)
HOMA-IR; mean (SD) [44]	5.9 (3.8)	7.1 (4.6)	6.1 (4.0)
**Somatic disorder**			
Diabetes mellitus type 2*;* n (%)	3 (2)	2 (6)	5 (3)
Impaired fasting plasma-glucose (6.1–6.9 mmol/l)*;* n (%) ^a^	5 (4)	0	5 (3)
Increased plasma-glucose (≥7 mmol/l)*;* n (%) ^a^	3 (2)	0	3 (2)
Hypercholesterolemia*;* n (%)	4 (3)	1 (3)	5 (3)
Hypertension*;* n (%)	3 (2)	1 (3)	4 (2)
Polycystic ovary syndrome*;* n (%)	13 (10)	N/A	N/A
Non-alcoholic fatty liver disease*;* n (%)	0 (0)	2 (6)	2 (1)
Obstructive sleep apnea*;* n (%)	0 (0)	0 (0)	0 (0)
≥1 of obesity-related metabolic disease*;* n (%) ^b^	19 (14)	7 (21)	26 (16)
Asthma*;* n (%)	28 (21)	7 (21)	35 (21)
Hypothyroidism*;* n (%)	10 (8)	1 (3)	11 (7)
**Micronutritional deficiencies (laboratory assessed)**			
Iron			
*Early functional iron deficiency* (transferrin saturation <16%)*;* n (%)	29 (22)	5 (16)	34 (22)
*Depleted stores* (serum-ferritin <12 μg/L or total iron binding capacity > 400 μg/dL)*;* n (%)	26 (20)	1 (3)	27 (17)
*Iron deficiency anemia* (depleted stores or early functional iron deficiency and Hb <120 g/L (men) or <130 g/L (women))*;* n (%)	0 (0)	0 (0)	0 (0)
Serum-cobalamine ^c^			
*Insufficiency* (<200 or <250 pmol/l)*;* n (%)	30 (24)	4 (13)	34 (22)
*Deficiency* (<100 or <150 pmol/l)*;* n (%)	10 (8)	2 (7)	12 (8)
Blood-folate deficiency (<305 nmol/l)*;* n (%)	10 (9)	0 (0)	10 (7)
Serum-25-OH-Vitamin D			
*Insufficiency* (25–50 nmol/l*);* n (%)	61 (46)	9 (32)	70 (45)
*Deficiency* (<25 nmol/l*);* n (%)	38 (29)	17 (52)	55 (35)
Serum-zinc deficiency (<10.7 πmol/l)*;* n (%)	10 (5)	1 (4)	11 (7)

Abbreviations: LDL, low density lipoprotein; HDL, high density lipoprotein; ALT, alanine transferase; HOMA-IR, Homeostatic model assessment-Insulin resistance; N/A, not applicable.

^a^ According to the definition by the World Health Organization [46].

^b^ Diabetes mellitus type 2, hypercholesterolemia, hypertension, polycystic ovary syndrome, non-alcoholic fatty liver disease, obstructive sleep apnea.

^c^ Reference values according to Beckman Coulter Inc (DxI, low cut-off) and Roche Diagnostics (Modular E120, high cut-off).

Seven percent (n = 11) had a systolic blood pressure ≥140 mmHg and 10% (n = 16) had a diastolic blood pressure ≥90 mm Hg, which, if attained twice, corresponds to the Swedish definition of systolic and diastolic hypertension respectively (when ≥16y) [47].

Forty-eight percent (n = 76) had at least one micronutritional deficiency, and 43% (n = 68) had at least one micronutritional insufficiency. Hypovitaminosis D was the most frequent deficiency (35%, n = 55). Nine participants with vitamin D-deficiency had increased serum-parathyroid hormone, however none of those had aberrations in plasma-calcium. A univariate analysis of variance was performed to evaluate the effects of seasonal variation on 25-OH-vitamin D with adjustment for skin type (dark/fair), age, gender and BMI. Mean summer levels were 42.9 nmol/l (SE = 2.0) and mean winter levels were 36.5 nmol/l (SE = 2.2), p = 0.032.

### Psychiatric disorders and medication, suicide attempts, mental health and health-related quality of life

**Table 3**displays frequencies of psychiatric disorders and medication, suicide attempts, and estimations of mental health and health-related quality of life. Sixteen percent (n = 26) were diagnosed with depressive episode and/or anxiety disorder, and 15% (n = 24) had at least one neurodevelopmental disorder, including ADHD (13%), Asperger’s disorder (3.6%), autistic disorder (1.2%) and Tourette’s disorder (0.6%). In terms of health-related quality of life scores, mental component scores were significantly lower (worse quality of life) than physical component scores (P<0.001).

**Table 3:** Psychiatric disorders, psychiatric medication, suicide attempts, mental health and health-related quality of life in n = 165 treatment-seeking young adults (16–25 y) with obesity.

Variable	Women (n = 132)	Men (n = 33)	Total (n = 165)
**Psychiatric disorder** ^**a**^			
Depressive episode*;* n (%)	21 (16)	1 (3)	22 (13)
Anxiety disorder*;* n (%)	16 (12)	4 (12)	20 (12)
ADHD-H/-I/-C*;* n (%)	14 (11)	7 (21)	21 (13)
Dyslexia*;* n (%)	24 (18)	9 (27)	33 (20)
Other neurodevelopmental disorders*;* n (%)	5 (4)	3 (9)	8 (5)
Eating disorder not otherwise specified*;* n (%)	2 (2)	0 (0)	2 (1)
≥1 Psychiatric disorder present*;* n (%) ^b^	37 (28)	10 (31)	47 (29)
Life-time history of ≥1 psychiatric disorder*;* n (%) ^b^	47 (36)	10 (30)	57 (35)
**Psychiatric medication**			
Antidepressants*;* n (%) ^c^	19 (14)	1 (3)	20 (12)
Psychostimulants*;* n (%) ^d^	9 (7)	4 (12)	13 (8)
Sedatives*;* n (%) ^e^	10 (8)	0 (0)	10 (6)
Sleeping medication*;* n (%) ^f^	11 (8)	4 (12)	15 (9)
For any psychiatric disorder*;* n (%) ^b^	27 (21)	8 (24)	35 (21)
**Suicide attempts, participant-reported**			
*Never;* n (%)	114 (86)	31 (94)	145 (88)
*>1 year ago;* n (%)	14 (11)	1 (3)	15 (9)
*<1 year ago;* n (%)	4 (3)	1 (3)	5 (3)
**HADS-Anxiety subscale [29],** total score; mean (SD)	8.0 (4.6)	6.9 (4.5)	7.8 (4.6)
*Normal (score ≤7);* n (%)	68 (52)	19 (58)	87 (53)
*Subclinical (score 8 to 10);* n (%)	28 (21)	7 (21)	35 (21)
*Clinical (score ≥11);* n (%)	35 (27)	7 (21)	42 (26)
**HADS-Depression subscale [29],** total score; mean (SD)	5.3 (4.3)	5.1 (3.6)	5.3 (4.1)
*Normal (score ≤7);* n (%)	96 (73)	24 (73)	120 (73)
*Subclinical (score 8 to 10);* n (%)	21 (16)	7 (21)	28 (17)
*Clinical (score ≥11);* n (%)	15 (11)	2 (6)	17 (10)
**Rosenberg Self-Esteem Scale [31],** total score; mean (SD)	15.7 (7.0)	18.3 (5.9)	16.2 (6.9)
*Low self-esteem (score ≤15);* n (%)	58 (44)	11 (33)	69 (42)
**Obesity-related Problems Scale [33],** total score; mean (SD)	67.0 (27.0)	50.6 (29.2)	63.7 (28.1)
*Mild impairment (score ≤39);* n (%)	20 (15)	12 (36)	32 (19)
*Moderate impairment (score 40 to 59);* n (%)	32 (24)	10 (30)	42 (25)
*Severe impairment (score ≥60);* n (%)	80 (61)	11 (33)	91 (55)
**Positive screening for ADHD using ASRS-v1.1 [37]** *;* n (%)	51 (39)	10 (30)	61 (37)
**Short Form-36 Health Survey [35]**			
*Physical component score*; mean (SD)	46.0 (11.7)	44.7 (9.0)	45.7 (11.2)
*Mental component score*; mean (SD)	35.0 (14.0)	39.4 (13.3)	35.8 (13.9)

Abbreviations: ADHD, attention-deficit/hyperactivity disorder; -H, hyperactive-impulsive; -I, inattentive; -C, combined; HADS, Hospital anxiety and depression scale; ASRS, Adult ADHD Self-report Scale.

^a^ Psychiatric disorders subclassified as in Diagnostic and Statistical Manual of Mental Disorders, 4th Edition [20].

^b^ Dyslexia and insomnia were excluded from the analysis.

^c^ Serotonin reuptake inhibitor, serotonin noradrenaline reuptake inhibitor (ATC-code N06A).

^d^ Metamphetamine, amphetamine, atomoxetin (ATC-code N06B).

^e^ Benzodiazepine derivates, hydroxizine (ATC-code N05B).

^f^ Benzodiazepine related drugs, melatonin (ATC-code N05C).

Forty percent (n = 60) of the participants were classified as having been exposed to at least one adverse childhood experience. Absent parent (due to parent living abroad, parent´s substance abuse, parent´s bipolar disorder, or parent´s decision to stop seeing the child) was the most common adverse experience (n = 43, 26%).

### Regression analysis of anxiety and depression symptomatology

Multiple linear regression analysis (**Table 4**) showed that present anxiety symptomatology measured by Hospital Anxiety and Depression Scale was independently positively associated with pain and low self-esteem (R^2^ = 0.33, P<0.001), but not with BMI, cardiorespiratory fitness or obesity-related problems (all P≥0.13) when adjusted for gender, age, economic strain, hazardous alcohol consumption, insomnia, insulin resistance and social support. In terms of depressive symptomatology, there were independent associations for cardiorespiratory fitness, self-esteem and obesity-related problems (R^2^ = 0.38, P<0.001), but not for BMI or pain (both P≥0.36) when adjusted for the same variables as above.

**Table 4:** Multivariable regression analysis of factors independently associated with Hospital Anxiety and Depression Scale (HADS) in n = 165 treatment-seeking young adults (16–25 y) with obesity.

	Regression coefficient	95% CI	P-value
**HADS-Anxiety**			
**Body mass index**	-0.057	-0.22 to 0.10	0.48
**Cardiorespiratory fitness** ^**a**^	-0.12	-0.27 to 0.036	0.13
**Pain** ^**b**^	2.4	0.82 to 4.00	0.003
**Self-esteem** ^**c**^	-0.25	-0.37 to -0.13	<0.001
**Obesity-related problems** ^**d**^	0.014	-0.015 to 0.044	0.34
**HADS-Depression**			
**Body mass index**	-0.039	-0.18 to 0.099	0.57
**Cardiorespiratory fitness** ^**a**^	-0.18	-0.31 to -0.045	0.009
**Pain** ^**b**^	0.65	-0.74 to 2.03	0.36
**Self-esteem** ^**c**^	-0.23	-0.33 to -0.12	<0.001
**Obesity-related problems** ^**d**^	0.031	0.005 to 0.058	0.018

Covariates: gender, age, economic strain, hazardous alcohol consumption, insomnia Homeostasis Model Assessment-Insulin resistance; and social support (all P>0.19). Adjusted R^2^_HADS-Anxiety_ = 0.33, P = <0.001; R^2^_HADS-Depression_ = 0.38, P = <0.001.

^a^ Astrand´s test [25].

^b^ European Quality of Life-5 Dimensions [42].

^c^ Rosenberg Self-Esteem Scale [31].

^d^ Obesity-related Problems Scale [33].

**Fig 1:**
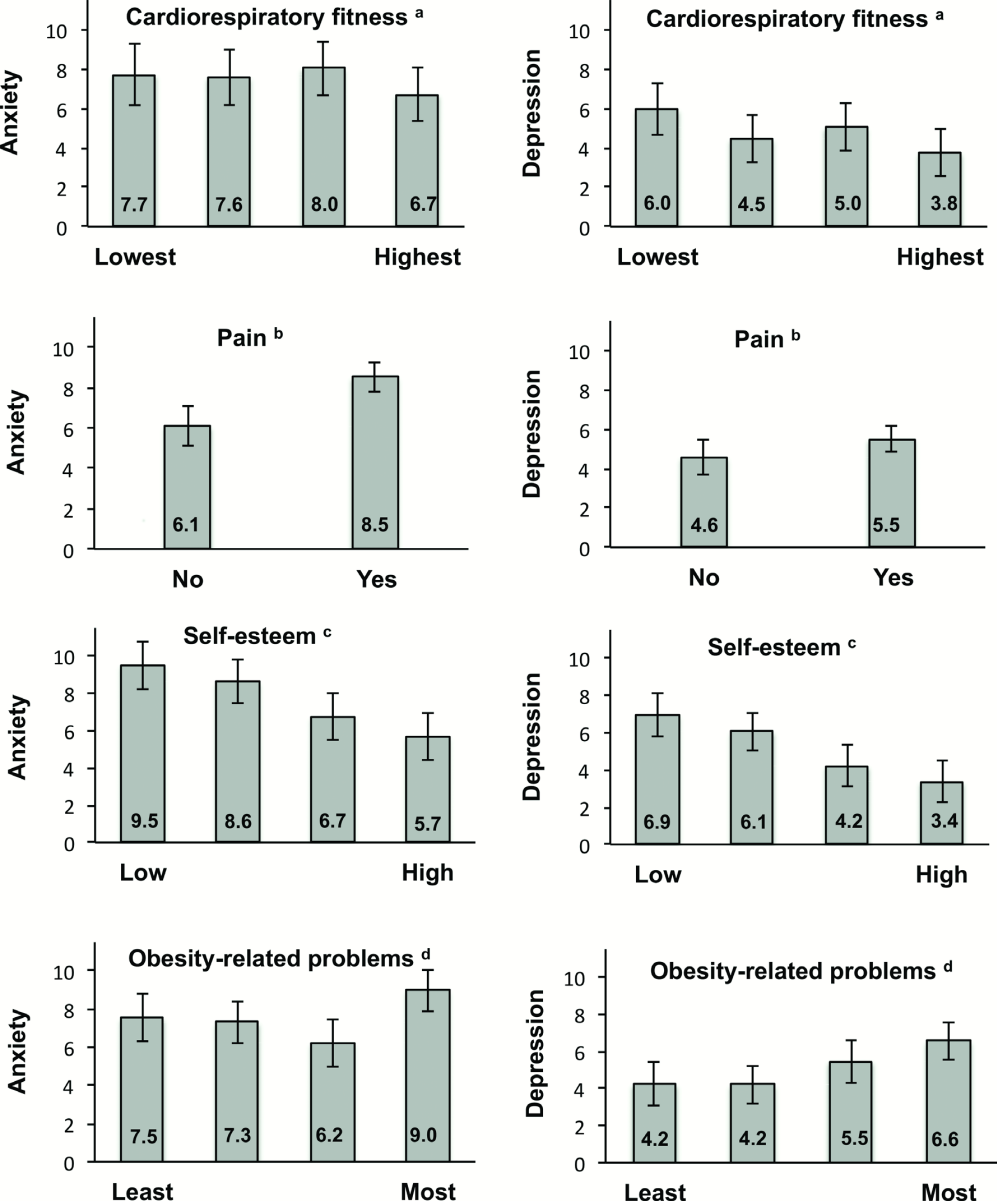
Estimated marginal means across quartiles of cardiorespiratory fitness, pain (yes/no), self-esteem and obesity related problems for anxiety and depression as measured by the Hospital Anxiety and Depression Scale (min-max: 0–21) in (n = 165) treatment-seeking young adults (16–25 y) with obesity. Values are mutually adjusted (except for cardiorespiratory fitness) for gender, age, body mass index, economic strain, hazardous alcohol consumption, insomnia, Homeostasis Model Assessment-Insulin resistance; and social support (all P>0.30). Error bars = 95% CI. ^a^ Astrand´s test [25]. ^b^ European Quality of Life-5 Dimensions [42]. ^c^ Rosenberg Self-Esteem Scale [31]. ^d^ Obesity-related Problems Scale [33].

**Fig 1**depicts estimated marginal means across quartiles of significantly associated variables for both anxiety and depressive symptomatology, adjusted for the same variables as above (except cardiorespiratory fitness).

## Discussion

### Main findings

In this cross-sectional study of treatment-seeking young adults with severe obesity, we found consistent indications of poor mental health. This included symptomatology of anxiety (47%), depression (27%) and ADHD (37%); low self-esteem (42%), attempted suicide (12%) and severe impairment in psychosocial functioning related to obesity (55%). Using Short Form-36 Health Survey, the mental component scores were significantly lower (mean 36) than the physical component scores (mean 46).

Psychiatric diagnoses were common (29% had one or more diagnosis), with depression and ADHD being the most frequent (both 13%). Six percent had other neurodevelopmental disorders (Asperger’s/autistic/Tourette´s disorder).

There was a high prevalence of risk factors for cardiometabolic disease, such as plasma lipid abnormality (62%), insulin resistance (82% according to Homeostatic model assessment-index) and poor cardiorespiratory fitness (92%). In terms of micronutritional status, 48% had at least one micronutritional deficiency, vitamin-D being the most common (35%).

Anxiety symptomatology was independently associated with low self-esteem and pain, whereas depressive symptomatology was associated with low self-esteem, low cardiorespiratory fitness, and obesity-related problems.

### Other research

Previous characterization studies have largely included either younger (children/teenagers) or older (adults) participants or generally focused on cardiometabolic risk factors [17, 18, 48]. The cross-sectional Teen-Longitudinal Assessment of Bariatric Surgery (Teen-LABS) Cohort characterized 15–18 year-olds, with a mean BMI of 50.5 kg/m^2^ prior to bariatric surgery [18]. Compared to the present study, the Teen-LABS-patients generally displayed higher rates of most cardiometabolic risk factors, which may be due to higher BMI than in the present study: hypertension (49%), impaired fasting glucose (26%), diabetes (14%) and dyslipidemia (50%). Sysko et al made a comprehensive evaluation of mental health in 200 teenagers undergoing bariatric surgery, and found, similar to the present study, 31.5% with axis I diagnoses [49].

Normative scores for HADS in Swedish young adults (16-23y) are 5.4 for anxiety and 3.0 for depression (7.8 and 5.3, respectively, in the present study population) [50]. Regarding SF-36, the same study group displays a mean physical component score of 55, and a mean mental component score of 46 (46 and 36, respectively, in the present study). A meta-analysis on use of the Rosenberg Self-Esteem scale in the U.S. found higher scores (better self-esteem) in18-22 year-olds than in the present study (22.3 compared to 16.2) [51]. Considering these data, the present study clearly indicates that the participants display clinically meaningful impairments in mental health compared to the general population (all weight categories).

Case-control studies of young adults (15–21 y) have found a higher prevalence of depression and anxiety in clinical obese participants (23% for depression and 40% for anxiety disorder) than in community samples of obese (9% for depression and 21% for anxiety) or normal-weight (10% for depression and 14% for anxiety) populations [52]. Quality of life is consistently lower in obese than normalweight adolescents (up to 20 y) with a clear positive linear relationship [53]. Suicidal ideation was found to be higher in extremely obese relative to healthy weight adolescents (OR: 2.3) [54]. Although there is a scarcity of studies on neurodevelopmental disorders in young adults with obesity, ADHD has been associated with obesity in adulthood (OR: 3.0) [55], and clinical populations of 16–20 year olds with autism or Asperger’s syndrome are more likely to be overweight or obese (odds ratios of 1.1 and 1.6 respectively) [56] than healthy control subjects.

Known psychiatric diagnoses in adolescents with obesity have been associated with program dropout as well as serious adverse events such as suicide and alcoholism after bariatric surgery [57]. The prevalence of depression tends to increase at about two years post-surgery [13], and worryingly, long-term follow-up (>24 months) of mental health consequences in adolescents and young adults after bariatric surgery is lacking [13].

The discrepancy of psychiatric diagnoses (depression/anxiety/ADHD) and corresponding symptomatology shown in this study may reflect underdiagnosis or prodromal symptomatology since psychiatric symptoms at this age are by no means definite and may develop into a wide array of psychiatric diseases [16]. ADHD-symptomatology such as impulsivity may also represent a specific trait of the young adulthood age period [7].

The associations found in the current study between anxiety and depressive symptomatology with pain, cardio-respiratory fitness, self-esteem and obesity-related problems, but to a lesser extent with BMI broaches the question of mediating variables in the associations between obesity and mental health. Preiss et al [58] found in a systematic review that depression was consistently associated with degree of obesity, education, body image, binge eating, physical health, physical activity, and psychological characteristics such as self-esteem and interpersonal effectiveness in obese individuals. Consequently the results from the present multiple regression analysis are partially in line with earlier studies. Less is known about associations between anxiety and obesity, but research suggests sleep [59], body esteem [60] and sedentary behavior as mediators [61].

### Strengths and limitations

The main strength of this study was the comprehensive assessments of variables associated with health and well-being in an understudied patient group. A majority of the participants were severely obese, which is increasing rapidly among adolescents [62, 63], and has been less studied than overweight and class I obesity.

The main limitation was the lack of a control group, limiting the possibilities to compare rates of health issues with normal weight peers or a non-treatment-seeking obese population. Control group comparisons are especially important since there has been a rise in mental health problems in Sweden among young adults during the last decades, measured by number of psychiatric diagnoses, hospitalizations and antidepressive medication use [64]. Moreover, 64% of participants were students (high school or higher education), possibly negatively affecting mental health [65], although we did not find this (data not shown). Finally, the cross-sectional design does not permit us to make any causal inferences.

There is a risk of socially desirable responses since the included participants were part of routine clinical care and could have responded in a way that allowed certain treatment options. The phenomenon of “impression management” has been found in adult participants in psychological assessment prior to bariatric surgery, resulting in false (underestimations) estimates of depressive symptoms [66]. Moreover, referrals to our clinic represent a highly selected population, meaning that the results may not be representative of young adults with obesity in the community.

Twenty percent (n = 46) of participants were men, which is in line with previous clinical obesity research [18]. This did not provide sufficient statistical power to analyze gender differences. Gender differences in enrolment may be due to women´s higher awareness of being overweight and proneness to seek weight loss treatment compared to men [67]. Moreover, we neither included questionnaires on eating disorders, nor assess personality disorders.

### Implications for future treatment, including bariatric surgery

The high prevalence of a wide array of mental health issues among treatment-seeking obese young adults displayed in the present study highlights the importance of making a thorough mental health assessment prior to treatment in order to not miss diagnoses and cognitive difficulties that may impact treatment outcome. Furthermore, we suggest careful re-evaluations since seemingly mild psychiatric signs at this age may develop into serious manifestations.

Young adults in general are less worried about future health hazards than older adults. Therefore, cardiometabolic co-morbidities of obesity, which tend to occur later in life, may be of little motivational value for a young adult compared to an older adult [68]. Targeting other outcomes, such as improved self-esteem or other factors associated with mental health in obese individuals may be a more successful way of boosting motivation [69]. Acceptance commitment therapy can also be used, with preliminary positive results on weight loss in adults compared to controls [70].

The high presence of poor mental health in combination with cardiometabolic risk factors and malnutrition should make young adults with obesity a prioritized patient category in the overall public health system. Young adults may, however, be the least likely age group to be treated for obesity [71], possibly due to failed transition of care, or perceived pessimism from clinicians that young adults with obesity can be successfully treated.

The pessimism surrounding young adults in conventional obesity treatment is increasingly pushing this age category towards bariatric surgery, despite a lack of evidence on both effectiveness and safety for this age group. Given the high frequency of mental health problems and malnutrition found in the current study, we urge caution since such complications may worsen after bariatric surgery, as well as predicting weight recidivism [72, 73].

Considering the high risk of surgery [12, 14, 15, 74], the long exposure for this age group, as well as the difficulty of reversing the surgery; balanced against the risk of worsening obesity and co-morbidity status, the severely obese young adult presents the clinician with a substantial dilemma. More evidence on the optimal treatment approach for this vulnerable population is therefore urgently needed.

## Conclusion

A wide range of health issues, including quite severe mental health problems, was prevalent in treatment-seeking young adults with severe obesity. The study provides the practitioner with a comprehensive characterization and highlights the vulnerability of this hitherto understudied patient category. Their overall poor health status is likely to constitute a major treatment challenge, including options relating to bariatric surgery. Further research is needed on how these prevalent and often severe mental health issues impact behavioral and bariatric treatment outcomes in young adults with severe obesity.

## Supporting Information

S1 Table Methodology for separate questions included in the questionnaire. (DOCX)

S2 Table Thresholds for metabolic risk values and micronutritional deficiencies measured by blood chemistry. (DOCX)
