# Supplementary material for: Who is the Treatment-Seeking Young Adult with Severe Obesity: A Comprehensive Characterization with Emphasis on Mental Health
Source: PLoS One. 2015 Dec 22;10(12):e0145273. doi: 10.1371/journal.pone.0145273 (PMC4687938; doi:10.1371/journal.pone.0145273)
Supplement: S1 Table — (DOCX) [file pone.0145273.s001.docx]

**S1 Table. Methodology for separate questions included in the questionnaire.**

| Variable | Question | Response alternatives |
| --- | --- | --- |
| **Nationality ^a^** | Country of birth: | Free text |
| **Mother´s nationality ^a^** | What is your parents’ country of birth? Mother: Father: | Free text |
| **Father´s nationality ^a^** | Father´s country of birth? | Free text |
| **Occupation** | What is your main occupation? | Permanent employment/ Temporary employment/  Business owner/  Sickness benefit last >30 days/Disability pension/ Student/Parental leave/  Unemployed/other |
| **Economic strain** | Did you experience any difficulties coping with private expenditures during the last year? | No/Yes, several times/Yes, once |
| **Sickness leave** | How many days were you on sickness leave last year? | Free text |
| **Tobacco smoking** | Did you ever smoke tobacco daily during at least six months? | Yes/no |
| **Cannabis** | Did you ever use cannabis? | No/Yes |
| **Alcohol ^b^** | What is your alcohol consumption/week? | Free text in numbers of units (glasses of alcohol). |
| **Sexuality** | How would you define your sexual orientation? | Heterosexual/Homosexual/Bisexual/Unsure |
| **Social support** | Do you know of any person whom you may get personal support from in case of a private crisis? | Yes, always/Yes, mostly/ No, mostly not/No, never |

**^a^** Responses were classified into Sweden/Europe outside Sweden/Africa/the Middle East, Asia, South America.

**^b^** Hazardous drinking was defined according to Swedish standards as weekly consumption of 14 units for men and 9 units for women, or intensive consumption of 5 units for men and 4 units for women. One unit equals 12 grams of 100% alcohol (1).

**References**

1. Alkohol och hälsa. En kunskaps-översikt om alkoholens positiva och negativa effekter på vår hälsa. Stockholm: 2005.
