# Supplementary material for: Who is the Treatment-Seeking Young Adult with Severe Obesity: A Comprehensive Characterization with Emphasis on Mental Health
Source: PLoS One. 2015 Dec 22;10(12):e0145273. doi: 10.1371/journal.pone.0145273 (PMC4687938; doi:10.1371/journal.pone.0145273)
Supplement: S2 Table — (DOCX) [file pone.0145273.s002.docx]

**S2 Table. Thresholds for metabolic risk values and micronutritional deficiencies measured by blood chemistry.**

| Variable | | Cut-off values | |
| --- | --- | --- | --- |
| **Fasting p-glucose ^a^ (1)** | |  | |
|  | Normal | <6.1 mmol/L | |
|  | Impaired | 6.1 to 6.9 mmol/L | |
|  | Increased | ≥7 mmol/L | |
| **b-HbA1c (2, 3)** | |  | |
|  | Intermediate risk | 42-47 mmol/mol | |
|  | Indicative of diabetes mellitus | ≥48 mmol/mol | |
| **HOMA-IR ^b^ (4)** | | <18 years: >3.16, ≥18 years: >2.5 | |
| **Total p-cholesterol ^c^ (5)** | | <20 years: ≥5.18 mmol/l, ≥20 years: ≥5.83 mmol/l | |
| **p-LDL-cholesterol ^c^ (5)** | | <20 years: ≥3.37 mmol/l, ≥20 years: ≥4.15 mmol/l | |
| **Fasting p-triglycerides ^d^ (5)** | | <20 years: ≥1.47 mmol/l, ≥20 years: ≥1.69 mmol/l | |
| **p-HDL-cholesterol ^c^ (5)** | | ≤1.04 mmol/l | |
| **p-ALT ^e^ (6, 7)** | | Women: | <18 years: >0.37 µkat/L, ≥18 years: ≥ 0.76 µkat/L |
|  |  | Men: | <18 years: >0.42 µkat/L, ≥18 years: ≥1.20 µkat/L |
| **Iron ^f^ (8)** | |  | |
|  | Early functional iron deficiency | Transferrin saturation <16 % | |
|  | Depleted stores | s-ferritin <12 µg/L or total iron binding capacity > 400 μg/dL | |
|  | Iron deficiency anemia | Depleted stores or early functional iron deficiency and Hb <120 g/L (men) or <130 g/L (women) | |
| **Vitamin D (9)** | |  | |
|  | Insufficiency | s-25-OH-vitamin D 25-50 nmol/l | |
|  | Deficiency | s-25-OH-vitamin D 25 <25 nmol/l | |
| **Folate (10)** | | b-folate <305 nmol/l | |
| **Vitamin B12** ^g^ | |  | |
|  | Insufficiency | s-cobalamine ^g^ <200 or <250 pmol/l | |
|  | Deficiency | s-cobalamine ^g^ <100 or <150 pmol/l | |
| **Zink (8)** | | s-zinc <10.7 πmol/l | |

Abbreviations: p-, plasma; b-, blood; s-, serum; HOMA-IR, Homeostatic model assessment-Insulin resistance; LDL, low density lipoprotein; HDL, high density lipoprotein; ALT, alanine aminotransferase.

^a^ To convert to mg/dl; divide by 0.05551 (11).

^b^ Fasting insulin (µU/ml) × fasting glucose (mmol/l)/22.5 (12).

^c^ To convert to mg/dL; multiply by 38.6 (5).

^d^ To convert to mg/dL; multiply by 88.6 (5).

^e^ To convert to IU/l; divide by 0.01667 (13).

^f^ S-ferritin was multiplied by 0.65 if high sensitive p-CRP >2 mg/L, to adjust for inflammation (14, 15).

^g^ Reference values according to Beckman Coulter Inc (DxI, low cut-off) and Roche Diagnostics (Modular E120, high cut-off).

**References**

1. Alberti KG, Zimmet PZ. Definition, diagnosis and classification of diabetes mellitus and its complications. Part 1: diagnosis and classification of diabetes mellitus provisional report of a WHO consultation. Diabetic medicine : a journal of the British Diabetic Association. 1998;15(7):539-53.

2. Nowicka P, Santoro N, Liu H, Lartaud D, Shaw MM, Goldberg R, et al. Utility of hemoglobin A(1c) for diagnosing prediabetes and diabetes in obese children and adolescents. Diabetes Care. 2011;34(6):1306-11.

3. Standards of medical care in diabetes--2013. Diabetes Care. 2013;36 Suppl 1:S11-66.

4. Keskin M, Kurtoglu S, Kendirci M, Atabek ME, Yazici C. Homeostasis model assessment is more reliable than the fasting glucose/insulin ratio and quantitative insulin sensitivity check index for assessing insulin resistance among obese children and adolescents. Pediatrics. 2005;115(4):e500-3.

5. Expert panel on integrated guidelines for cardiovascular health and risk reduction in children and adolescents: summary report. Pediatrics. 2011;128 Suppl 5:S213-56.

6. Schwimmer JB, Dunn W, Norman GJ, Pardee PE, Middleton MS, Kerkar N, et al. SAFETY study: alanine aminotransferase cutoff values are set too high for reliable detection of pediatric chronic liver disease. Gastroenterology. 2010;138(4):1357-64, 64.e1-2.

7. Mårtensson A. Reference intervals for haematology analytes. Preliminary results from the Nordic Reference Interval Project (NORIP). . Klinisk Biokemi I Norden. 2003(15):20 - 1.

8. Institute of Medicine Panel on M. Dietary Reference Intakes for Vitamin A, Vitamin K, Arsenic, Boron, Chromium, Copper, Iodine, Iron, Manganese, Molybdenum, Nickel, Silicon, Vanadium, and Zinc. Washington (DC): National Academies Press (US)

Copyright 2001 by the National Academy of Sciences. All rights reserved.; 2001.

9. Ross AC, Manson JE, Abrams SA, Aloia JF, Brannon PM, Clinton SK, et al. The 2011 report on dietary reference intakes for calcium and vitamin D from the Institute of Medicine: what clinicians need to know. J Clin Endocrinol Metab. 2011;96(1):53-8.

10. Institute of Medicine Standing Committee on the Scientific Evaluation of Dietary Reference I, its Panel on Folate OBV, Choline. The National Academies Collection: Reports funded by National Institutes of Health. Dietary Reference Intakes for Thiamin, Riboflavin, Niacin, Vitamin B6, Folate, Vitamin B12, Pantothenic Acid, Biotin, and Choline. Washington (DC): National Academies Press (US)

National Academy of Sciences.; 1998.

11. Fonseca V. Clinical Diabetes: Saunders; 2006.

12. Matthews DR, Hosker JP, Rudenski AS, Naylor BA, Treacher DF, Turner RC. Homeostasis model assessment: insulin resistance and beta-cell function from fasting plasma glucose and insulin concentrations in man. Diabetologia. 1985;28(7):412-9.

13. Schiff R, Maddrey W, Sorrell M. Schiffs disease of the liver: Wiley-Blackwell; 2011.

14. Thurnham DI, McCabe LD, Haldar S, Wieringa FT, Northrop-Clewes CA, McCabe GP. Adjusting plasma ferritin concentrations to remove the effects of subclinical inflammation in the assessment of iron deficiency: a meta-analysis. The American journal of clinical nutrition. 2010;92(3):546-55.

15. Nead KG, Halterman JS, Kaczorowski JM, Auinger P, Weitzman M. Overweight children and adolescents: a risk group for iron deficiency. Pediatrics. 2004;114(1):104-8.
